# Influence of Grains Shape Irregularity in Porous Ceramics—Numerical Study

**DOI:** 10.3390/ma13081944

**Published:** 2020-04-21

**Authors:** Danuta Miedzińska

**Affiliations:** Faculty of Mechanical Engineering, Military University of Technology, Kaliskiego 2 St.,00-908 Warsaw, Poland; danuta.miedzinska@wat.edu.pl

**Keywords:** porous ceramics, structure, physical properties

## Abstract

The presented study deals with the analysis of the stochastic geometry of grains on ceramic foam strength behavior. A microstructural finite element (FE) model of a grainy structure of such a material was developed and stochastic changes to the grain geometry (initially of a regular cubic shape) were introduced. The numerical compression test of a series of finite element models was carried out with the use of LS Dyna computer code. To consider the ceramic specific behavior, the Johnson Holmquist constitutive model was implemented with parameters for alumina. The influence of the stochastic irregularities on the ceramic foam strength was observed—the geometry changes caused an increase in the maximum stress, which could be the basis for the indication that the production of the energy absorbing material should be based on mostly irregular grains.

## 1. Introduction—Aim of Study

Solid ceramic materials are well known and studied. Their behavior is analytically described, and this description was successfully applied in numerical methods: the finite element method (FEM) [1], meshless [2], and more. Constitutive material models were developed for solid ceramics. The most popular is Johnson Holmquist, which was used in the presented study and described below.

Porous ceramics are a fairly new group of materials. They were patented in 1963 [3], however the first studies were presented in 2001 [4]. Nevertheless, new methods of design, production and applications are developed worldwide. Porous ceramics are characterized by [5,6]:

- good chemical stability—the possibility of various base materials and processing selection allows for the preparation of structures resistant to different working conditions, e.g., corrosion;
- high specific strength and rigidity—the base material of a porous structure is stiff ceramic, which causes gas or liquid pressure, and other stress loadings do not influence the shape and the size of pores;
- good thermal stability—heat-resistant porous ceramics are able to filtrate the molten steel or high-temperature burning gas.

The above-mentioned advantages show the wide possibility to apply porous ceramics, for example, in various industries, such as chemical, energy, metallurgy, electronic engineering and design [7].

**Figure 1:**
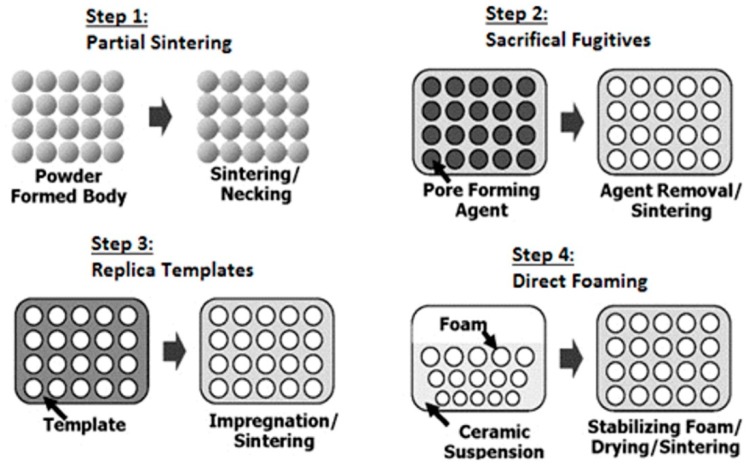
Fabrication processes of macroporous ceramic [8].

Porous ceramics can be characterized with pores classified into three classes depending on the pore diameter, *d*: macroporous (*d* > 50 nm), mesoporous (50 nm > *d* > 2 nm) and microporous (*d* < 2 nm) [8]. Owing to this fact, various methods of production were developed. For macroporous foams, the representative production process can be described in four steps: partial sintering, sacrificial fugitives, replica templates and direct foaming (Figure 1) [8].

**Figure 2:**
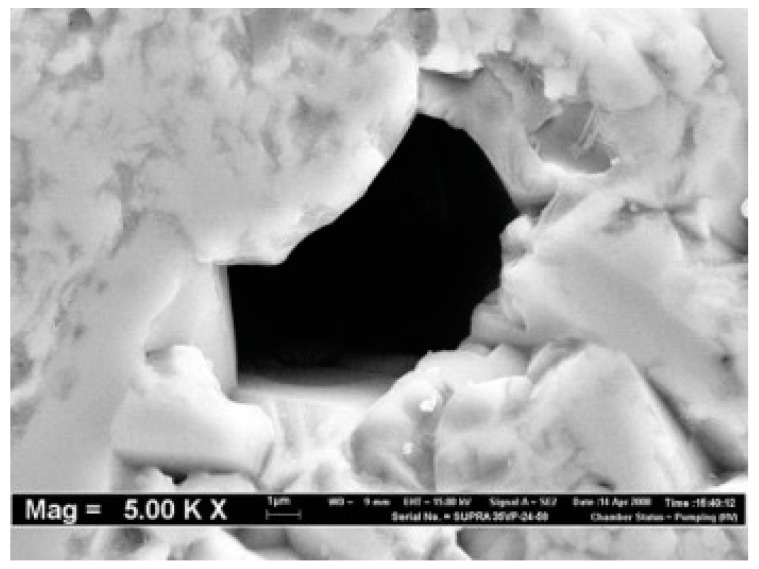
An illustration of microporous ceramic foams: a single open pore in Al_2_O_3_ ceramic microstructure magnification [11].

The most interesting ceramic structures discussed in the paper are that with meso and micro pores which appear between ceramic grains. Such structures, obtained through sintering, are observed and presented in literature [9,10,11] (Figure 2).

**Figure 3:**
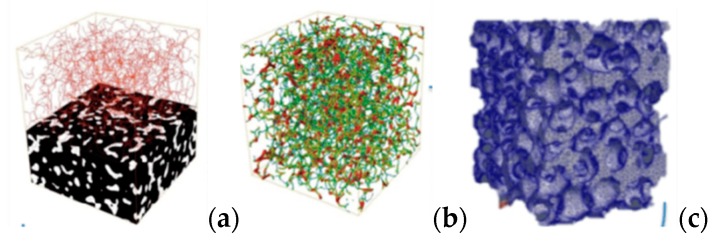
An example of the modeling process of ceramic foam real structure: (**a**) computed tomography results marked as black and white bitmaps and thresholding of the pores, (**b**) pore space stl model, (**c**) finite element model [13].

Some attempts to analyze porous ceramic structure can also be found in literature. The most popular method is using an X-ray tomography to develop a numerical model of a real structure [12,13], however, such studies do not allow for the checking of the general rules which can be useful in designing materials with desired properties. An example of such a method is given in Figure 3.

**Figure 4:**
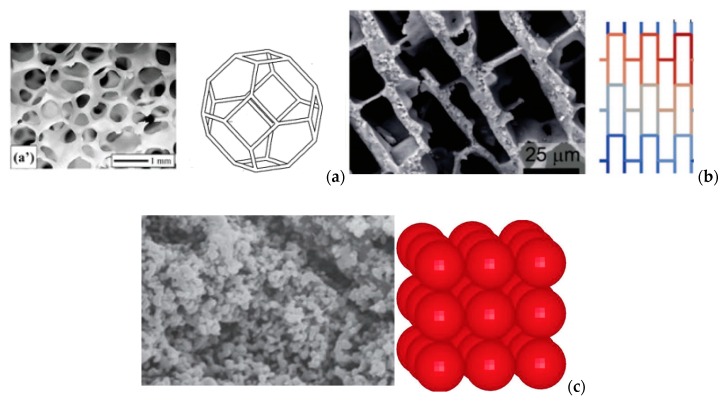
Examples of idealistic models of ceramic foams (**a**) using Kelvin structure, (**b**) beams [14] (**c**) and spheres [15].

Also, some idealistic models were developed using, e.g., Kelvin tetrakaicecahedron, beams [14] or spheres [15], as seen in Figure 4.

On the basis of the presented methods of ceramic structure analyzing, it was concluded that the influence of a grain stochastic shape which influences on the foam mechanical properties was not concerned. The real structure models do not allow us to study the phenomena in every kind of structure. The selected region can only be observed, and some conclusions can be drawn for the locally observed phenomena. The idealistic models allow for the observation of some general rules of the foam behavior, however, they do not consider the stochastic parameters typical for a ceramic microstructure, such as the variable size or shape of grains.

It was decided that we would develop a numerical model which allows for the study of the random geometry of grains in accordance with their mechanical parameters. The proposed method of analyzing the random ceramic structure can be assumed as novel, in accordance with the presented literature review and lack of such analyses.

In this paper, the influence of stochastic changes on the geometry of grains, which builds the ceramic foam microstructure, was studied with the use of the finite element method.

## 2. Numerical Model Development and Convergence Analysis

**Figure 5:**
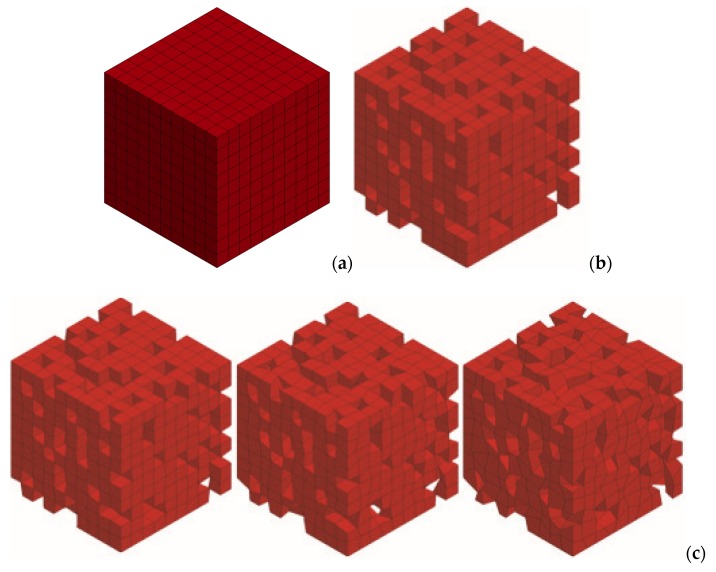
Examples of developed numerical models: (**a**) Step 1—solid model, (**b**) Step 2—porous model with grains of a regular shape, (**c**) Step 3—porous model with grains of a stochastic shape (radius of a sphere for node translation—0.1, 0.2 and 0.3).

**Figure 6:**
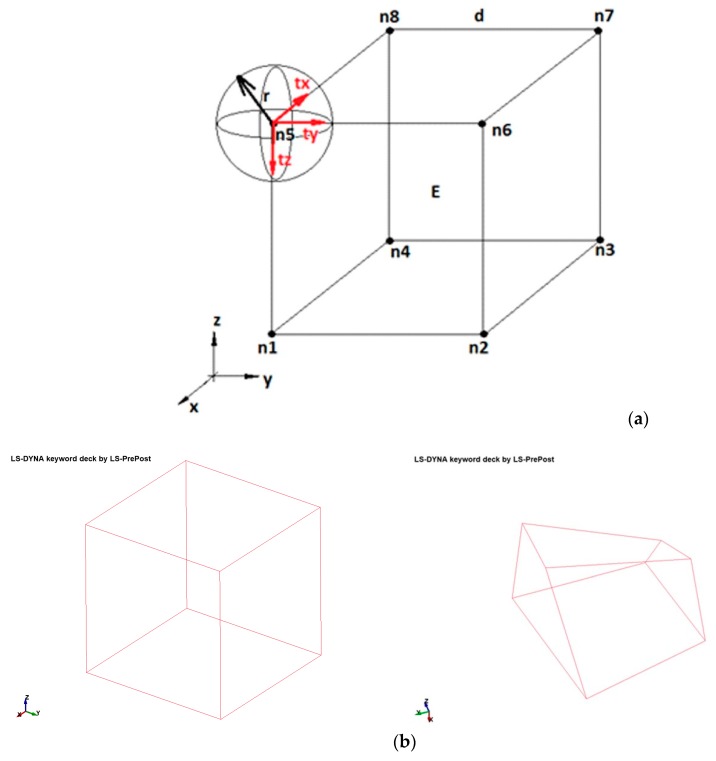
(**a**) Scheme of stochastic translation of modes: E—element, n1÷n8—nodes, x,y,z—coordinates, d—distance between nodes, r—radius of a sphere in which translation is allowed, tx, ty, tz—translation vectors in x, z and z directions; (**b**) example of the transformed element.

The microstructural model of the porous ceramic material was built from solid hexagonal elements. A series of stochastic models was developed in the following steps:

Step 1: a solid model built from 10 × 10 × 10 brick elements was built (Figure 5a). Each element reflects a grain;

Step 2: the porosity of the model was achieved by drawing 20% of the elements and removing them from the model built in Step 1. Step 2 was performed ten times, and ten different models were developed (Figure 5b);

Step 3: the stochastic process of changing the elements (grains geometry) was introduced. A FEM input file was converted in Visual Basic for Applications (VBA)—the coordinates of the nodes of elements were stochastically changed. The nodes were translated within a sphere with a radius of 0.1, 0.2 and 0.3 initial distance between the neighboring nodes. A translation value for each coordinate was stochastically drawn (using Excel RAND function). The node translation method was presented in Figure 6, and the developed model was shown in Figure 5c. It should be mentioned that the correction of the porous space volume was introduced to achieve the same volume in each model. Ten models for each radius for each model prepared in step 2 were developed—300 models were built in total.

A finite element study was carried out with the use of LS Dyna software (v971, Livermore Software Technology Corp., Livermore, CA, USA), which is dedicated to dynamic phenomena studies. A central difference scheme was applied in the system for the explicit time integration to assesses the linear change in acceleration. It was developed based on a single degree of freedom damping system, in which forces acting on mass *m* are as follows: *f_s_*—elastic force, *f_I_*—inertia force, *f_D_*—damping forces, *p*(*t*)—external forces [16].

The equations of equilibrium are based on d’Alambert’s principle:

(1) $$f_{I}+f_{D}+f_{int}=p\left(t\right)\;f_{I}=m\ddot{u},\;\ddot{u}=\frac{d^{2}u}{dt^{2}}-\mathrm{acceleration}\;f_{D}=c\dot{u},\;\dot{u}=\frac{du}{dt}-\mathrm{velocity}\;f_{int}=ku,\;u-\mathrm{displacement}$$

In the equations presented above, *m* is mass, *k* is the linear stiffness and *c* is a damping coefficient.

The equations of motion for the linear behavior lead to a linear ordinary differential equation:

(2) $$m\ddot{u}+c\dot{u}+ku=p\left(t\right)$$

where *p*(*t*) is assumed as the loading force. It should be observed that for the nonlinear case, the internal force varies as a nonlinear function of the displacement, leading to the nonlinear formula:

(3) $$m\ddot{u}+c\dot{u}+f_{int}\left(u\right)=p\left(t\right)$$

Analytical solutions for linear ordinary differential equations are known, therefore, a dynamic response of a linear system subjected to the harmonic loading can be also considered.

The closed form solution can be defined as:

(4) $$\left(t\right)=u_{0}\cos\omega t+\frac{{\dot{u}}_{0}}{\omega }\sin\omega t+\frac{p_{0}}{k}\cdot \frac{1}{1-\beta^{2}}\left(\sin\bar{\omega }t-\beta \sin\omega t\right)$$

with the initial conditions: initial displacement $u_{0}$, initial velocity ${\dot{u}}_{0}$ and static displacement $\frac{p_{0}}{k}$. In Equation (4), the following symbols were used:

- $\omega =\sqrt{\frac{k}{m}}$—circular frequency for a single degree of freedom,
- $p_{0},\bar{\omega }$—amplitude and frequency in the harmonic loading equation $P\left(t\right)=p_{0}sin\bar{\omega }t$,
- $\beta =\frac{\bar{\omega }}{\omega }$—applied load frequency.

For nonlinear problems, only numerical solutions are possible. In the problem described in the paper, the explicit central difference scheme, built in LS Dyna, was applied for the equations of motion integration.

To describe the central difference method, the semi-discrete equations of motion at time *n* are defined as:

(5) $$Ma^{n}=P^{n}-F^{n}+H^{n}$$

where *M* is the diagonal mass matrix, *P^n^* accounts for external and body force loads, *F^n^* is the stress divergence vector and *H^n^* is the hourglass resistance. To advance time *t^n^*^+1^, the central difference time integration is used in the following form:

(6) $$a^{n}=M^{-1}\left(P^{n}-F^{n}+H^{n}\right)$$

(7) $$v^{n+\frac{1}{2}}=v^{n-\frac{1}{2}}+a^{n}\Delta t^{n}$$

(8) $$u^{n+\frac{1}{2}}=u^{n}+v^{n+\frac{1}{2}}\Delta t^{n+\frac{1}{2}}$$

where:

(9) $$\Delta t^{n+\frac{1}{2}}=\frac{\Delta t^{n}+\Delta t^{n+1}}{2}$$

and *v* and *u* are the global nodal velocity and displacement vectors, respectively. The geometry can be updated by adding the displacement increments to the initial geometry:

(10) $$x^{n+1}=x^{0}+u^{n+1}$$

For the purpose of finite element mesh development, 4- nodal brick elements were used. MAT_JOHNSON_HOLMQUIST_CERAMICS was applied as a material model. This model is useful in the analysis of brittle materials, such as ceramics, subjected to large pressures, shear strain and high strain rates. The model considers the phenomena that appear when brittle materials are subjected to load and damage. The ceramic-type material equivalent stress can be calculated as [16]:

(11) $$\sigma^{*}=\sigma_{i}^{*}-D\left(\sigma_{i}^{*}-\sigma_{f}^{*}\right)$$

where:

(12) $$\sigma_{i}^{*}=a{\left(p^{*}+t^{*}\right)}^{n}\left(1+c\;\ln{\dot{\varepsilon }}^{*}\right)$$

describes the intact undamaged state. The damage parameter:

(13) $$D=\sum \frac{\Delta \varepsilon^{p}}{\varepsilon_{f}^{p}}$$

represents the accumulated damage based on an increase in the plastic strain per a computational cycle and the plastic strain to fracture:

(14) $$\varepsilon_{f}^{p}=d_{1}{\left(p^{*}+t^{*}\right)}^{d_{2}}$$

and

(15) $$\sigma_{f}^{*}=b{\left(p^{*}\right)}^{m}\left(1+c\;ln{\dot{\varepsilon }}^{*}\right)\leq SFMAX$$

is the damaged state. In each case, “*” indicates a normalized quantity, the stresses are normalized by the equivalent stress at the Hugoniot elastic limit, the pressures by the pressure at the Hugoniot elastic limit and the strain rate by the reference strain rate [16]. The material constants for Al_2_O_3_ applied in the model are presented in Table 1.

The boundary conditions applied in the FE model were set to reflect the dynamic compression test. The numerical sample was placed on the rigid wall. The compression was carried out with the moving rigid wall (velocity *v* = 1 mm/ms). The automatic single surface contact, considering a penalty function, was set. The static friction coefficient between the ceramic faces and the rigid wall and ceramic structure was 0.2 and dynamic coefficient was 0.1.

Firstly, the convergence analysis of the developed model was carried out. The methods for such analyses are known [18,19]. The most popular method is to use the experimental data to compare them with calculations. However, this method cannot be used in the case of the proposed study. The data that can be found in literature refer to the compression tests of ceramic foams treated as macrostructures in which only the porosity and grain size (sieve analysis of powder) are considered as a factor influencing the mechanical properties [20,21,22]. Those studies do not allow for their comparison to the results of the presented study.

**Figure 7:**
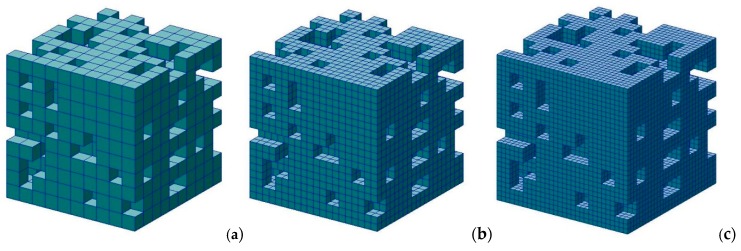
Models prepared for a mesh size convergence study (**a**) one element per grain, (**b**) eight elements per grain, (**c**) 27 elements per grain.

We decided to check the model convergence using the mesh size and eratio analysis. For the first one, the exemplary models of both regular and irregular grains were prepared by applying 1, 8 and 27 elements per one grain (Figure 7). The results of the FE tests are presented in Table 2 showing the maximum stress and time of analysis. It should be noted that the maximum stress appeared for the same value of strain for each model. It can be observed that the model with one element reflecting one grain can be used. The maximum stress parameter is the most important factor for the analysis presented in the paper.

The next step is to check the model convergence, which was the eratio analysis. Eratio is the energy factor that is applied in LS Dyna to verify the energy conservation rule. The energy ratio is considered as [16]:

(16) $$eratio=\frac{E_{total}}{E_{total}^{0}+W_{ext}}$$

where $E_{total}$ is the total energy in the considered time step, $E_{total}^{0}$ is the initial total energy and $W_{ext}$ is external work. In all analyzed models, eratio was equal to 1 ± 3% during the analysis.

## 3. Results and Discussion

**Figure 8:**
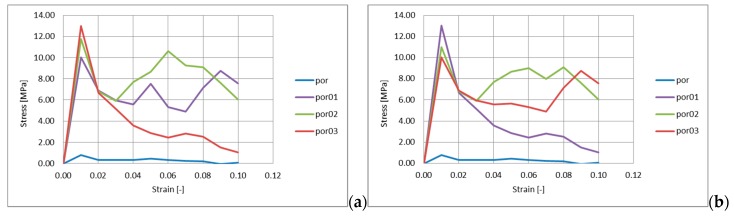
Results of compression tests of models with a porous ceramic microstructure: por—regular structure, structure with nodes displacement within a sphere of with a radius of 0.1 (por01), 0.2 (por02) and 0.3 (por03) of element edge: (**a**) regular behavior, (**b**) irregular behavior.

The results were presented as stress–strain charts. The engineering stress was calculated as a ratio of the reaction force from the stationary rigid wall to the initial cross section of the sample. The engineering strain was calculated as a ratio of the loading rigid wall displacement to the initial height of the sample. The examples of such charts are presented in Figure 8.

**Figure 9:**
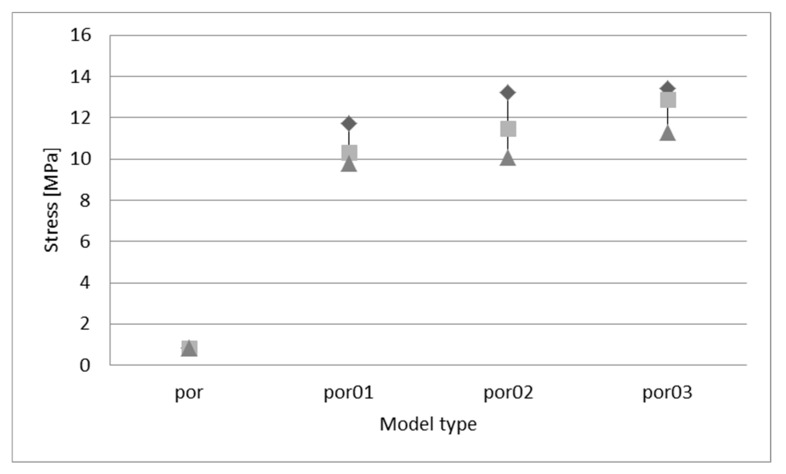
Stress vs. model type chart: ■—average value, ♦—maximum value, ▲—minimum value.

The reason behind presenting two exemplary charts in Figure 8 is that some irregularities appeared in a testing series of models, and to show this difference more clearly. Firstly, it should be observed that the change in geometry caused a significant increase in the maximum stress (first peak stress). However, the differences were not obvious. In Figure 8a, the trend is quite understandable—the bigger displacement for nodes is assessed, and the bigger stress value is achieved, however, in Figure 8b this rule is not restricted. To understand this phenomenon, a chart with average, maximum and minimum values of the maximum stress was prepared (Figure 9). In this chart, it can be observed that the trend of the irregularity increase causes an increase in stress appearance. Nevertheless, it can be observed that, sometimes, the stress can be lower for a higher irregularity.

**Figure 10:**
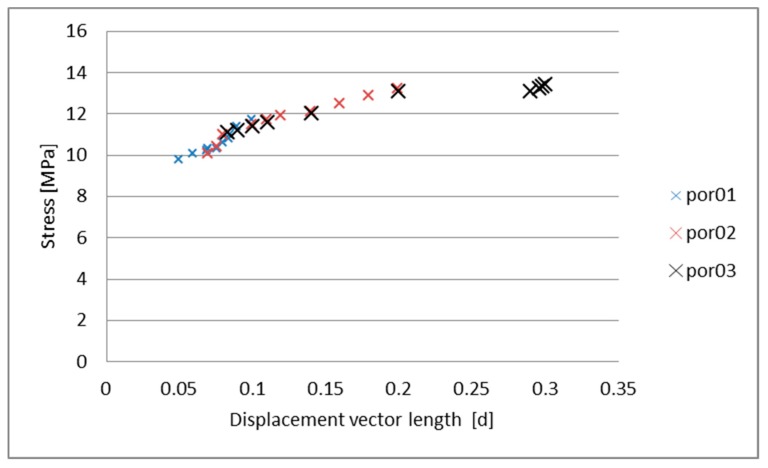
Stress vs. averaged displacement length.

As already mentioned, the model was developed using random drawings of node displacement that need to be placed in the sphere with the described radius. The averaged translation vector length was calculated and analyzed. The calculations of the maximum stress value were set as a function of the average value of nodes displacement length and presented in Figure 10. A linear trend can be observed, however, there are some deviations.

**Figure 11:**
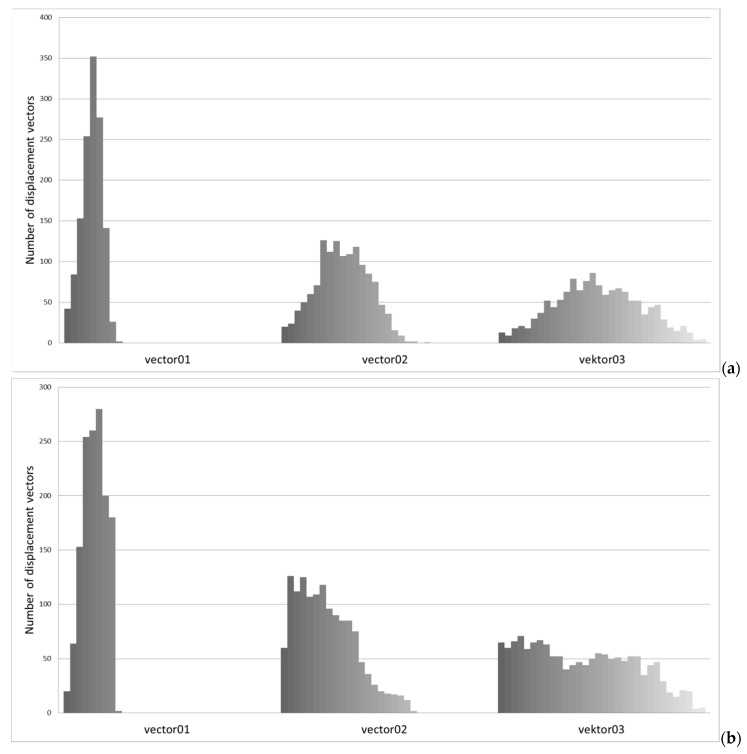
Number of node displacement vectors counted within the selected ranges for the models analyzed in: (**a**) Figure 7a, (**b**) Figure 7b.

The reason for these deviations could be the displacement vector on the base of stochastic drawing and, in some models, a different number of shorter and longer vectors. For example, the drawing for por0.3 model can result in vector lengths comparable to those for 0.1 model, which can cause the differences shown in Figure 8a,b. Therefore, it was decided to develop additional charts to verify those assumptions. The charts of the number of node displacement vectors counted within the selected ranges for the models analyzed in Figure 8 are presented in Figure 11. Based on those results, the assumption presented above was confirmed. The distribution of vector length can influence the strength characteristic.

**Figure 12:**
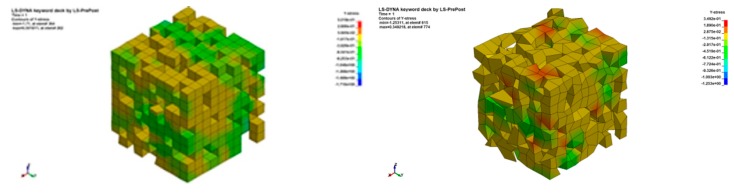
Y—stress distribution for regular and irregular structure.

Eventually, the stress distribution was observed in the model (Figure 12). Interestingly, the irregularity in the shape of grains caused an increase in the stress concentration regions, which could be the reason for the maximum stress increase in comparison to regular structures. Such a concentration appeared in vertices of the irregular elements. The more deformed the element was, the greater stress concentration appeared.

The presented study considers the influence of the stochastic changes in porous ceramics grain geometry on ceramic foam mechanical properties. The following conclusion can be drawn: an increase in the irregularity of grains can cause a significant change in the strength behavior of the ceramic foam. The design and production of a new material with the desired properties can be based on this observation. This phenomenon is particularly useful when the first maximum stress determining the energy absorption and effectiveness of protection is used, e.g. during a gun bullet impact. The foam production process can begin from the selection of grains of the most irregular shapes within the selected size. However, the presented analysis is limited to some extent, as it only considers the grains of one sieve class size.

## Figures and Tables

**Table 1:** Material constants for Al_2_O_3_ [17].

Parameter	Unit	Value
Density	kg/m^3^	3.226
Shear modulus	GPa	90.16
Strength constants		
A	-	0.93
B	-	0.31
C	-	0.0
M	-	0.6
N	-	0.6
EPSI (Ref strain rate)	-	1.0
Tensile strength	GPa	0.2
HEL	GPa	2.79
HEL pressure	GPa	1.46
HEL volumetric strain	-	0.01117
HEL strength	GPa	2.0
Damage constants		
D1	-	0.005
D2	-	1.0
Equation of state constants		
K1 – bulk modulus	GPa	130.95
K2	GPa	0
K3	GPa	0
Beta	-	1.0

**Table 2:** Mesh size convergence study results for the regular model.

Mesh Size	Average Maximum Stress [MPa]	Analysis Time
1 element per grain,	10.3	10 min
8 elements per grain,	10.3	38 min
27 elements per grain	10.3	2 h 4 min
